# Combined 3D-QSAR, Molecular Docking and Molecular Dynamics Study on Derivatives of Peptide Epoxyketone and Tyropeptin-Boronic Acid as Inhibitors Against the β5 Subunit of Human 20S Proteasome

**DOI:** 10.3390/ijms12031807

**Published:** 2011-03-09

**Authors:** Jianling Liu, Hong Zhang, Zhengtao Xiao, Fangfang Wang, Xia Wang, Yonghua Wang

**Affiliations:** 1 College of Life Sciences, Northwest University, Xi’an, Shaanxi 710069, China; 2 Center of Bioinformatics, Northwest A&F University, Yangling, Shaanxi 712100, China; E-Mail: dcpwyh@163.com; 3 School of Chemical Engineering, Dalian University of Technology, Dalian 116012, China

**Keywords:** ubiquitin-proteasome, 3D-QSAR, CoMFA, CoMSIA, homology modeling, molecular docking, molecular dynamics

## Abstract

An abnormal ubiquitin-proteasome is found in many human diseases, especially in cancer, and has received extensive attention as a promising therapeutic target in recent years. In this work, several *in silico* models have been built with two classes of proteasome inhibitors (PIs) by using 3D-QSAR, homology modeling, molecular docking and molecular dynamics (MD) simulations. The study resulted in two types of satisfactory 3D-QSAR models, *i.e.*, the CoMFA model (Q^2^ = 0.462, R^2^_pred_ = 0.820) for epoxyketone inhibitors (EPK) and the CoMSIA model (Q^2^ = 0.622, R^2^_pred_ = 0.821) for tyropeptin-boronic acid derivatives (TBA). From the contour maps, some key structural factors responsible for the activity of these two series of PIs are revealed. For EPK inhibitors, the N-cap part should have higher electropositivity; a large substituent such as a benzene ring is favored at the C6-position. In terms of TBA inhibitors, hydrophobic substituents with a larger size anisole group are preferential at the C8-position; higher electropositive substituents like a naphthalene group at the C3-position can enhance the activity of the drug by providing hydrogen bond interaction with the protein target. Molecular docking disclosed that residues Thr60, Thr80, Gly106 and Ser189 play a pivotal role in maintaining the drug-target interactions, which are consistent with the contour maps. MD simulations further indicated that the binding modes of each conformation derived from docking is stable and in accord with the corresponding structure extracted from MD simulation overall. These results can offer useful theoretical references for designing more potent PIs.

## 1. Introduction

Ubiquitin-proteasome system (UPS), as a major factor in regulated intracellular proteolysis in eukaryotic cells, is essential to maintain intracellular protein homeostasis and control important signaling pathways [1,2]. In the proteolytic process, the destined degradation protein is first labeled with a poly-ubiqutin chain through a cascade of enzymes (E1, E2, E3), then the ubiquitylated protein is recognized and degraded by the 26S proteasome, a multicatalytic multisubunit protease complex [3]. Two types of 26S proteasome have been identified, *i.e.*, constitutive proteasome and immunoproteasome [4].

The 26S proteasome consists of a 20S core particle (20S CP) and one or two 19S regulatory particles (19S RP) capped at either (or both) ends of the core [5]. X-ray crystallography studies [6–9] have demonstrated that 19S RP is built of a ring shaped base and a lid-structure, that regulates the entrance of substrate to the attached 20S proteasome [10], the 20S CP is a conserved hollow cylinder-shaped structure which is composed of four homologous rings, arranged in the sequence α_7_β_7_β_7_α_7_. Each β ring contains three distinct catalytic activities with three different subunits, namely chymotrypsin-like (β5), trypsin-like (β2) and the post-glutamyl peptide hydrolyzing, or caspase-like (β1) [11–13]. All three peptidases exhibit the same catalytic mechanism, in which the N-terminal threonine residue is the active nucleophile [14,15]. Among them, the importance of individual subunit activities for proteasomal function is as follows: β5 >> β2 ≥ β1 [16].

Owing to the significance of the proteasome, it is not surprising that aberrations and deregulations of the proteasome contribute to the pathogenesis of several human diseases. Thus, proteasome inhibitors (PIs) have become an attractive agent for human diseases therapy, especially for cancer [17–22]. Their anti-inflammatory and anti-cancer effects are particularly achieved through inhibiting activation of the transcription factor NFκB and promotion of apoptosis in rapidly dividing cells [23–25]. The most important class of PIs is the peptidic inhibitors, such as aldehydes [1], boronates [26], *etc*. Because of the highly reactive functional aldehyde group, aldehydes lack specificity; bortezomib, a famous PI of the boronate inhibitor family, has been approved for treatment of multiple myeloma and mantle cell lymphomas by the FDA [27]. However, some side effects of bortezomib have also been reported [28]. Thus, it is urgent to develop more potent, selective and clinic-friendly PIs. Epoxyketones (α, β-ketoepoxides) (EPK) are the second-generation PIs found in the late 90s [29,30]. Unlike other PIs, epoxyketones show potent selectivity to the proteasome, without inhibitory effects on other proteases such as calpain, trypsin, papain, chymotrypsin and cathepsins [31], due to the fact that epoxyketone moiety can form a morpholino adduct with the active site amino terminal Thr of the β5 subunit [32]. Epoxyketone inhibits primarily the CT-L activity of the proteasome [33], but also the T-L and C-L activities with slower rates [31]. Another attractive class of PIs is boronic acid derivatives of tyropeptin (TBA). Tyropeptins A, produced by *Kitasatospora sp.* MK993-dF2, is novel proteasome inhibitor [34]. Takumi Watanabe and coworkers also synthesized a set of TBA derivatives, exhibiting potent inhibitory against the CT-L activity of human proteasome [35].

Quantitative structure activity relationship (QSAR), which quantitatively correlates the variations in biological activity with the properties or molecular structures, is one of the most effective approaches for designing new chemical identities and understanding the action mechanisms of drugs [36–38]. In recent years, great attention has been paid to discovery and synthesis of novel PIs, studies regarding QSAR of existing PIs is still relatively insufficient although some 3D-QSAR models of PIs have been reported [39,40]. The authors offered useful information about the binding mode between the inhibitors and the proteasome through ligand-based model. However, detailed insights into the active site are still unclear, since the X-ray crystallographic structure of the human proteasome has not been reported to date. Thus, in order to reveal the structural features of inhibitors of the β5 subunit of human proteasome, a set of *in silico* methods including 3D-QSAR, homology modeling, molecular docking and molecular dynamics simulations have been conducted on EPK and TBA in the present work. As far as we know, this study presents the first 3D-QSAR study for these two kinds of PIs, which will provide detailed information for understanding these two series of compounds and aid screening and design of novel inhibitors.

## 2. Materials and Methods

### 2.1. Data Sets

All potent inhibitors of β5 subunit of the human proteasome used in the present study are collected from recent literatures [35,41]. Discarding compounds with undefined inhibitory activity or unspecified stereochemistry, 45 compounds of EPK and 41 compounds of TBA are employed in this work. Each group of compounds is divided into a training set for generating the 3D-QSAR models and a testing set for evaluating the 3D-QSAR models at a ratio of 4:1. The compounds in the test set have a range of biological activity values similar to that of the training set. Their IC_50_ values are converted into pIC_50_ (*i.e*., −logIC_50_) values and used as dependent variables in the CoMFA and CoMSIA calculation. The pIC_50_ values of the EPK and TBA compounds cover an interval of three and four log units, respectively. The structures of these two groups and their IC_50_ and pIC_50_ values are given in Appendix.

### 2.2. Molecular Modeling and Alignment

The 3D-QSAR study and molecular docking is performed using SYBYL 6.9 (Tripos, Inc). The 3D structures of all compounds are constructed using the Sketch Molecule function with Sybyl software. The geometry optimization of all compounds is carried out by using the TRIPOS force field with the Gasteiger-Huckel (GH) charges, and repeated minimization is performed using Powell conjugated gradient algorithm method with convergence criterion of 0.05 kcal/mol Å. Of note, molecules containing boron atoms are not supported by SYBYL because it does not provide the force field parameters for the boron atom in default settings. For this reason, we changed the boron atom in TBA derivatives to a carbon atom ‘C.3’ in SYBYL according to the strategy adopted in the literatures [42] and colored it with ‘magenta’ for distinction.

**Figure 1.**
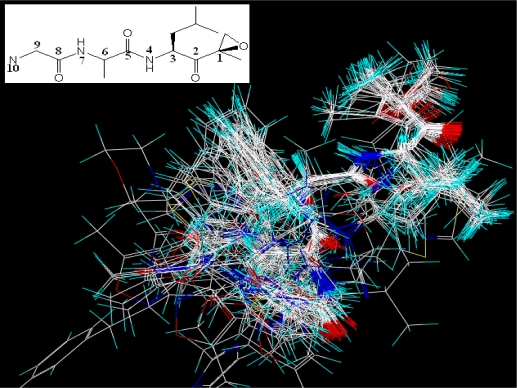
Superimposition of EPK compounds in the training and test sets with common substructure shown in the upper left corner.

**Figure 2.**
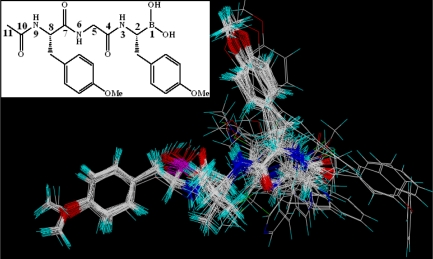
Superimposition of TBA compounds in the training and test sets with common substructure shown in the upper left corner.

Structure alignment is considered as one of the most critical step in CoMFA and CoMSIA analysis, so the template molecule selection for alignment and the alignment methods are crucial to the CoMFA and CoMSIA models. The most active compound of each group is used as a template for superimposition, which is assumed to represent the most bioactive conformation. The common fragments of EPK inhibitors and TBA inhibitors shown in the upper left corner of Figure 1 and Figure 2, respectively, is selected for database alignment rules in SYBYL command. Other molecules in the data set are superimposed on it (shown in Figure 1 for EPK and Figure 2 for TBA compounds).

### 2.3. Calculation and Selection of Molecular Descriptors

Molecular descriptors are quantitative representations of the structural and physicochemical feathers of molecules, which have been extensively used in the SAR studies [43–46]. In the present work, the DRAGON (http://www.talete.mi.it/index.htm) is employed to compute the molecular descriptors based on the MOL2 format of all minimized molecules. The program contains scripts for generating 1664 parameters of different types including: Constitutional, Topological, Geometrical, Charge, GETAWAY (Geometry, Topology and Atoms-Weighted AssemblY), WHIM (Weighted Holistic Invariant Molecular descriptors), 3D-MoRSE (3D-Molecular Representation of Structure based on Electron diffraction), Molecular Walk Counts, BCUT descriptors, RDF (Radial Distribution Functions), 2D Autocorrelations, Aromaticity Indices, Randic Molecular Profiles, Functional Groups, Atom-Centered Fragments, Empirical and Properties [47]. The stepwise linear regression method as the variable selection in R software (www.r-project.org) is used to obtain the best relevant and meaningful descriptors. The obtained descriptors are further put into the partial least squares analysis for building more reasonable QSAR models.

### 2.4. 3D-QSAR Studies

CoMFA and CoMSIA analyses are performed to construct good predictive QSAR models and to analyze the effect of each field on the activities of PIs. Models of steric and electrostatic fields for CoMFA are based on both Lennard-Jones and Coulombic potentials [48]. A 3D grid box of 2.0 Å is created around the aligned molecules. Steric and electrostatic energies are calculated using a sp3 carbon atom probe with a charge of +1.00 and van der Waals radius of 1.52 Å at each intersection lattice point of the grid. The CoMFA cutoff values of steric and electrostatic fields are set to 50 kcal/mol for EPK and 30 kcal/mol for TBA, respectively. Other parameters are set as default. The CoMFA fields are automatically scaled by the CoMFA-STD method in SYBYL.

The CoMSIA method defines explicit hydrophobic, hydrogen bond (H-bond) donor and acceptor descriptors in addition to the steric and electrostatic used in CoMFA. CoMSIA fields calculations are performed by constructing the same lattice boxes as those used in CoMFA calculations. A sp3 carbon probe atom is used to calculate each field with a charge of +1.00, a radius of 1.00 Å, hydrophobicity +1.00, and H-bond donor and acceptor property +1. The attenuation factor is set to a default value of 0.3 for these two classes of PIs. CoMSIA similarity indices (AF) for molecule *j* with atom *i* at grid point *q* are calculated by the following formula (1):

(1) $$A_{F,k}^{q}(j)=-\sum \omega_{\text{probe},k}\omega_{ik}e^{-\alpha r_{iq}^{2}}$$

where *k* represents the steric, electrostatic, hydrophobic, or hydrogen-bond donor or acceptor descriptor. A Gaussian type distance dependence is used between the grid point *q* and each atom *i* of the molecule.

The partial least squares (PLS) analysis is used to derive the 3D-QSAR models by constructing a linear correlation between the CoMFA/CoMSIA (independent variables) and the activity values (dependent variables). To select the best model, the cross-validation (CV) analysis is performed using the leave-one-out (LOO) method in which one compound is removed from the data set and its activity is predicted using the model built from rest of the data set [49]. The sample distance PLS (SAMPLS) algorithm is used for the LOOCV. The optimum number of components used in the final analysis is identified by the cross-validation method. The Cross-validated coefficient Q^2^, which as statistical index of predictive power, is subsequently obtained. To evaluate the real predictive abilities of the CoMFA and CoMSIA models derived by the training set, biological activities of an external test set is predicted. The predictive ability of the model is expressed by the predictive correlation coefficient R^2^_pred_, which is calculated by the following formula (2):

(2) $${\text{R}}_{\text{pred}}^{2}=(\text{SD}-\text{PRESS})\;/\;\text{SD}$$

Where SD is the sum of squared deviations between the biological activities of the test set and mean activity of the training set compounds, PRESS is the sum of squared deviations between experimental and predicted activities of the test set compounds.

### 2.5. Homology Modeling

For the rational design of new drugs, structural information about the target protein and specifically binding ligands is of utmost importance [50]. Due to the unavailability of the X-ray structure of human proteasome, the homology modeling for the protein structure from its primary sequence is performed. The target protein is the β5 subunit of human proteasome whose amino acid sequence (ID CAA64838.1) is obtained from the NCBI Web site (http://www.ncbi.nlm.nih.gov). The template protein (PDB entry code 1G65 chain K, resolution: 2.25 Å) identified by Blast Search (http://www.ncbi.nlm.nih.gov/BLAS) is employed here for the construction of the 3D model of the target protein. Its crystal structure is downloaded from Brookhaven Protein Database (http://www.pdb.org/pdb/home/home.do).

**Figure 3.**
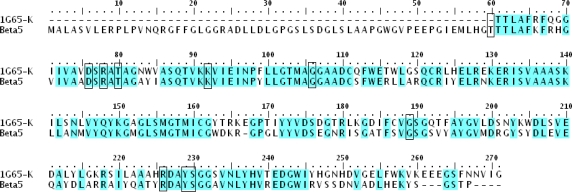
Alignments of the sequences of 1G65 chain K template and β5 target protein. Cyan color regions denote that the amino acid residues in the individual column are identical in the sequence alignment. The dashed lines denote the amino acid residues deletion. Key binding site residues are highlighted in black rectangles.

The initial sequence alignment of the target and template sequences is carried out using the ClustalW program [51], and the sequence identity obtained is 68% (shown in Figure 3). The resulting alignment is subsequently submitted to SWISS-MODEL server (http://swissmodel.expasy.org/) [52–54] for a comparative structural modeling. For molecule docking purpose, all hydrogen atoms are subsequently added to the unoccupied valence of heavy atoms at the neutral state (pH = 7.0) using the biopolymer module of SYBYL package.

### 2.6. Molecular Docking

To understand the detailed interaction of the β5 subunit of human proteasome with its inhibitors and to develop 3D-QSAR models, molecular docking analysis is carried out using the Surflex, which combines Hammerhead’s empirical scoring function with a molecular similarity method (morphological similarity) to generate putative poses of ligand fragments [55]. Two parameters, *i.e.*, protomol_bloat and protomol_threshold, determine how far a potential ligand should extend outside of the concavity and how deep into the protein the atomic probes are used to define the protomol. Michael Groll and coworkers have reported that the crystallized waters are of importance in mediating the interactions between the epoxomicin (ligand) and the terminal Thr of β5 subunit (1G65_K) [32]. Thus, our docking analysis is performed as follows: (1) The target protein structure is aligned with the template protein, then the cocrystalized ligand (EPX) and water molecules of 1G65_K are merged into the corresponding sites of the target protein structure; (2) The template 1G65_K is removed, while the original crystallized waters and ligand are retained; (3) To EPK, the protomol is generated using a ligand approach considering the ligand and water molecules with the specified 1_0.55 of bloat and threshold; to TBA, an automatic approach considering the water molecules with specified 1_0.46 of bloat and threshold is applied to generate the protomol. Meanwhile, the resulting homology protein structure is further developed using the protein preparation and refinement utility provided by SYBYL. During docking processes, the protein is considered rigid, and the ligand molecules are flexible. When the docking run is finished, it affords the top 10 docking poses of each ligand ranked by total scores.

In addition, for validating whether the crystallized waters are significant, the docking studies are also conducted in absence of the crystallized water molecules in the same conditions as mentioned above.

### 2.7. Molecular Dynamics Simulations

MD simulations are conducted with Amber 10 [56], utilizing the 3D structure of the docked complex with compound 11 of EPK and compound 2 of TBA as starting conformations, respectively. The general atom force field (GAFF) [57] and the AMI-BCC [58] method are employed to set the two ligand’s parameters and charges via antechamber module of Amber 10. Standard AMBER force field for bioorganic systems (ff99SB) [59] is chosen to depict the protein parameters. The initial conformers are neutralized by adding sufficient Cl^−^ counterions and solvated in a same size rectangular box (71.55 × 93.08 × 77.68 Å^3^) of TIP3P water [60], both with a minimum solute-wall distance of 12 Å. The cut-off distance for computing the nonbonded interactions is truncated at 10 Å; long-range electrostatic interactions are calculated using the particle-mesh-Ewald (PME) method [61] with default values. SHAKE [62] is applied to all bonds involving H-atoms.

Prior to MD simulations, each system is energetically minimized with the complex atoms constrained to eliminate possible bad contacts through 2500 steepest descent steps and another 2500 conjugate-gradient steps. Following that, MD simulations commence by heating up the systems to 300 K at a constant force of 2.0 kcal/mol Å^−2^ constraining the protein atoms. Then, a 50 ps of density equilibrated is applied at 300 K with the complex atoms constrained. After that, the system is equilibrated with a collision frequency of 1 ps^−1^ at a constant temperature and pressure. Finally, two 5 ns MD simulations are performed with a 2 fs time step at the isothermic-isobaric (NPT) canonical ensemble and under the periodic boundary conditions. The total number of the atoms in each simulation systems is 42,400 and 42,410 including complex and waters.

## 3. Results and Discussion

### 3.1. 3D-QSAR Models

The statistical parameters obtained from the best CoMFA and CoMSIA models according to ligand-based alignment and receptor-based alignment rules are listed in Table 1. The CoMFA models are constructed from steric and electrostatic descriptor fields, and the CoMSIA models are built by varying the steric, electrostatic, hydrophobic, and hydrogen-bond donor and acceptor descriptor fields. The Q^2^, SEP, SEE and F values are computed as defined in SYBYL. For analysis, we mainly focus on the best 3D-QSAR models derived from ligand-based CoMFA and CoMSIA methods for EPK and TBA, respectively.

From the pool of 1310 Dragon descriptors, two parameters were identified as statistically significant to the EPK inhibitory activity, *i.e.*, EEig04r and Mor24e. Another two descriptors (RDF050M and AlogP2) among 1293 are most relevant to TBA inhibitory activity. The molecular descriptors and definitions are shown in Table 2. These two groups of descriptors are further added to 3D-QSAR analysis for generating reliable models.

#### 3.1.1. EPK

In terms of EPK, its CoMFA model validated internally yields the Q^2^ = 0.462 with six optimum components. This model has a small SEE (0.202), suggesting that the CoMFA model is reliable and predictive. The steric and electrostatic fields contribute 41.8% and 42.9%, respectively. Therefore, these two fields have almost the same influence on the inhibitory activity of EPK. Furthermore, EEig04r and Mor24e contribute 10% and 5.3% to this model, showing that topological molecular characters and 3D-MoRSE descriptors also affect the EPK inhibitory activity. Without these two parameters, the model built by steric and electrostatic fields would be overfitting.

**Figure 4.**
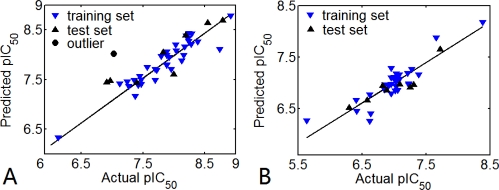
(**A**) Plot of predicted activities *versus* experimental activities for CoMFA analysis; (**B**) Plot predicted activities *versus* experimental activities for CoMSIA analysis. The solid lines are the regression lines for the fitted and predicted bioactivities of training and test compounds in each class.

During the cross-validation procedure, compound 25 is detected as an outlier (residual between the experimental value and predicted value is nearly to 1.0 log unit) for the CoMFA model. Some reasons may result in this appearance as an outlier. Compound 25 has a unique structure feature, which is different from other compounds. After removal of this compound, the predicted value changed from 0.680 to 0.820. The plot of the predicted *versus* actual pIC_50_ for the CoMFA analyses is shown in Figure 4(A). It can be seen that the data points are uniformly distributed around the regression line, indicating the reasonability of this model.

#### 3.1.2. TBA

For TBA, the optimal CoMSIA model validated internally yields Q^2^ = 0.622 with three optimum components. The small SEE (0.208) also indicates that this model is reliable and predictive. The steric, electrostatic, hydrophobic and H-bond acceptor field contributions are 0.035%, 0.117%, 0.122%, and 0.078%, respectively. From the contributions, the electrostatic and hydrophobic interactions of the ligand with the receptor are more important than the other two interactions to the inhibitory activity of TBA. The contributions of RDF050M and AlogP2 are 21.3% and 43.5%, respectively, showing that these two factors affect the TBA inhibitory activity dramatically. Formally, RDF code is based on the radial distribution function of an ensemble with N atoms, *i.e.*, probability distribution of finding atom on a sphere with radius *r* [63]. For the RDF050m descriptor, the sphere radius is 0.5 Å and the atomic weights are atomic masses (*m*). AlogP2 is the square of the Ghose-Crippen octanol-water coefficient (*AlogP*), which denote the hydrophobic/hydrophilic character of the molecule. These two descriptors reflect the importance of physicochemical and hydrophobility to their inhibitory activity.

The model is further validated using an external test set of eight compounds. Finally, agreeable statistical result (R^2^_pred_ = 0.821) is obtained for TBA. The plot of the predicted *versus* actual pIC_50_ values for the CoMSIA is shown in Figure 4(B). The well distribution of these data points around the regression line suggests that the model is reasonable.

### 3.2. 3D-QSAR Contour Map Analysis

**Figure 5.**
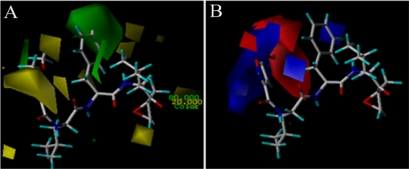
CoMFA StDev*Coeff contour maps of EPK. (**A**) steric field contour map (green: favored, yellow: disfavored); (**B**) electrostatic field contour map (red: disfavored areas of positive potential; blue: favored areas of positive potential). Compound 11 is shown in tubes as a reference.

**Figure 6.**
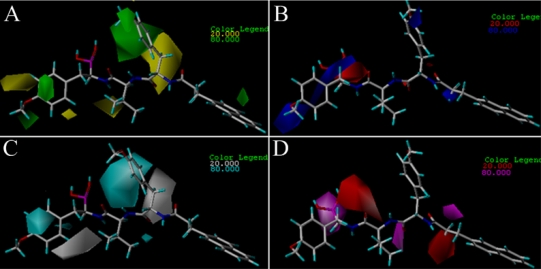
CoMSIA StDev*Coeff contour maps of TBA. (**A**) steric field contour map (green: favored; yellow: disfavored); (**B**) electrostatic field contour map (red: disfavored areas of positive potential; blue: favored areas of positive potential); (**C**) hydrophobic field counter map (cyan: favored; white: disfavored); (**D**) H-bond acceptor field counter map (magenta: favored; red: disfavored). Compound 2 is shown in tubes as a reference.

The greatest advantage of the 3D-QSAR modeling is the visualization of the results as 3D coefficient contour plots, which are helpful for us to further understand the nature of the receptor-ligand binding regions and the effects of these regions on steric, electrostatic, hydrophobic, H-bond donor and receptor fields for the biological activity. With consideration of both the internal and external predictive powers, the ligand-based CoMFA model for EPK is selected for each conformation to construct the stdev*coeff contour maps to view effects on the target features, while the ligand-based CoMSIA model is used for TBA. The maps generated depict regions having scaled coefficients greater than 80% (favored) or less than 20% (disfavored). For visualization, molecule 11 of EPK and molecular 2 of TBA is selected to demonstrate the contour maps (Figure 5 and Figure 6, respectively).

#### 3.2.1. EPK

In Figure 5A, a big yellow polyhedron upon the isoxazole ring and a small one close to the -MeOCH_2_ group at C3-position of the isoxazole ring suggest that bulky substituents in these areas will significantly decrease the biological activity. This is consistent with the reported experimental results. Compounds 3 and 10 with morpholine and isopropyl group at this position both have lower activity. Two small yellow polyhedrons near the methyl group at the C1-positon of the main chain and adjoining the epoxyketone indicate that large substituents in these areas will significantly decrease the biological activity. Contrarily, a big green polyhedron around the 4-, 5-position of the benzene ring indicates that more bulky substituents in this area may greatly increase the biological activity. This is confirmed by the fact that compounds 42 and 43, without a benzene ring, both have lower activity. In Figure 5B, a big red polyhedron and two small ones between the isoxazole ring and benzene ring suggest that positively charged substituents in this area will dramatically decrease the inhibitor activity. A big blue polyhedron beside the isoxazole indicates that positively charged substituents in this area will increase the inhibitor activity. Another two small blue polyhedrons upon the benzene ring and above the oxygen atom at the C8-position of the main chain also suggest positively charged substituents are favored for inhibitor activity. Compounds 39, 40 and 41 without negative charged substituents extending in red contour regions are observed to have lower biological activity. Compounds 23 and 27 without positive charged substituents extending in blue contour regions also have lower biological activity.

#### 3.2.2. TBA

In Figure 6A, a big yellow polyhedron area in the back of the C8-position of the main chain shows an unfavorable steric interaction in this position, which indicates that a bulky substituent in this area decreases the biological activity dramatically. This interprets why all compounds of this series do not carry large subsitituents. Two small yellow polyhedrons beside the isopropyl group and near the anisole group at the C2-position of the main chain suggest that bulk substituents in this position are not favored for inhibitor activity. The big green polyhedron around the anisole group at the C8-position of the main chain indicates that large substituent in this region have favorable steric interactions. Two small green polyhedrons in the anisole group suggest that large substituents are favored in this region. In Figure 6B, a red polyhedron area behind the B1-position of the main chain shows that electronegative groups are favored here. In contrast, the blue polyhedrons show the electropositive favored regions. There is one big blue region present beside the anisole group at the C2-position and two small blue regions present adjacent to the methyl group of anisole group at the C8-position of the main chain and around the ethyl group at the C11-position. In Figure 6C, two white polyhedrons show unfavorable hydrophobic interaction regions. One appeared in the back of the C8-position of the main chain, the other appeared in the left of isopropyl group. A big cyan polyhedron surrounding the anisole group at the C8-position and a small one upon the anisole group at the C2-position of the main chain indicate favorable hydrophobic interactions in these areas. In Figure 6D, a magenta polyhedron near the hydroxyl group at the B1-position and two small ones beside the isopropyl group and upon the ethyl group at the C11-position of the main chain indicate that these areas are favored for H-bond acceptor interactions, while another two red polyhedrons with one above the N3-position of the main chain and the other below the C11-position show disfavored regions for H-bond acceptor interactions.

### 3.3. Homology Modeling

Comparative or homology modeling is a methodology to predict protein structure based on the general observation that proteins with similar sequences have similar structures. Given an experimentally established protein structure (template), models can be generated for a homologous sequence (target) that shares either significant sequence (∼30% or more) or structural similarity with the template [64].

**Figure 7.**
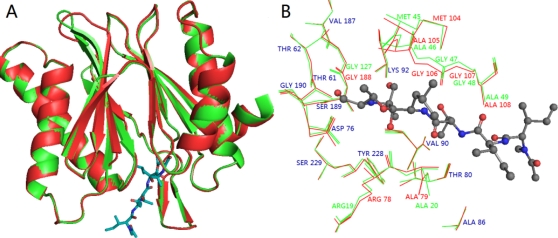
(**A**) The superposition of 1G65_K template (green ribbon) and the β5 subunit of human proteasome (red ribbon) from homology modeling; (**B**) The enlargement of the superposition structure of the active site with ligand EPX displayed as balls and sticks. The residues from the template protein and the homology modeling protein are highlighted in green and red colors, respectively. The same residues are labeled in blue color, while the different residues between them are labeled in their own color.

In the present work, the whole sequence identity between the target (β5 subunit of human proteasome) and the template protein (PDB code: 1G65_K) is 68%. Except the precursor amino acid sequence (amino acids 1–59), the functional sequence identity is 71% (amino acids 60–247). Thus, with a high level of sequence identity, the appreciated template 1G65 can be used to construct a reliable 3D structure and guarantee the quality of homology model. Since an N-terminal threonine (Thr) residue is very important for the catalytic activity (Thr60 in human), we added a Thr60 to the N-terminal of the modeling protein which we did not modeled by homology modeling. The superposition of the model to template is shown in Figure 7, indicating that the overall conformation of the modeling target is very similar to the template with a root-mean-square deviation (RMSD) of 1.423 Å (<2 Å). Furthermore, we carefully analyzed the alignment in the critical residues of the binding site and found that almost all important amino acids (such as Asp76, Thr80, Lys92, Gly106, Ser189 and Ser229) overlapped well in 3D space for the two structures (The amino-acid numbering in the sequences of template and modeling structures starts at the N-terminal catalytic threonines (Thr2 and Thr60)).

### 3.4. Docking Analysis and Comparison with 3D Contour Maps

Molecular docking for all 86 inhibitors was performed to find the optimal conformation of the ligands in the binding pocket of the β5 subunit of the human proteasome, and to understand the nature of interactions between them, as well as to complement the 3D-QSAR studies for the rational design of drugs.

The top ranked docked solution of each class was found in one favorable cluster of docking poses with an average RMSD value of 2.27Å and 2.54Å, respectively, demonstrating the binding mode is correctly reproduced [65]. By this performance, the binding modes for the most potent compound of each group exhibited statistically significant total score results of 9.41 and 9.44, respectively. In addition, some key residues such as Thr60, Arg78, Thr80, Lys92, Gly106, Ser189 and Ser229 appeared in the binding cavity, which further demonstrated the reasonability of docking protocol.

As we expect, the docking result of EPK in the absence of the crystallized waters is not good. The reason might be that crystallized waters are very important in mediating the interactions between the epoxomicin and terminal Thr of the β5 subunit (1G65_K), as described in the previous work by Michael Groll and coworkers [32]. Compared with the docking result with the crystallized waters, the total score of the most active compound is lower (6.23). And the correlation between the activity value and the score is bad (R^2^ = 0.0118). However, for TBA the docking result without the crystallized waters is almost the same as that with crystallized waters, indicating that the crystallized waters have little effect on the interaction between TBA and the β5 subunit.

#### 3.4.1. EPK

**Figure 8.**
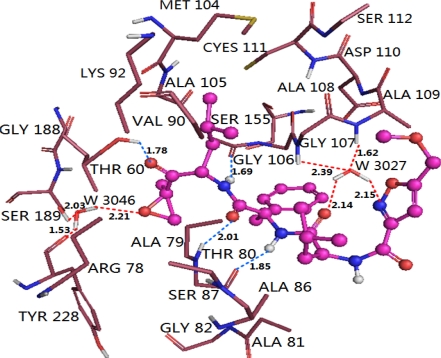
Docked conformations derived for molecule 11 of EPK (shown in ball and stick model) with the β5 subunit of human proteasome. H-bonds formed between residues and molecule directly and mediated by water indirectly are shown as dotted lines with blue and red color, respectively. W3027 and W3046 represent water molecules. The nonpolar hydrogen atoms are removed for clarity.

The binding mode of compound 11, shown in Figure 8, is taken as an example for analysis. The ligand core is anchored in the binding site via four H-bonds and two water-mediated contacts with the protein. The oxygen at C5-position of the main chain forms a H-bond with the backbone NH of Thr80 (–O···HN, 2.01 Å, 152.4°), and the oxygen at C2-position of the main chain forms a H-bond with the side chain of Thr60 (-O···HO, 1.78 Å, 131.9°). While the two hydrogen atoms at the N4- and N7- positions of the main chain form two H-bonds with the backbone carboxyl group of Gly106 (-O···HN, 1.69 Å, 146.5°) and the backbone carbonyl group of Thr80 (–O···HN, 1.85 Å, 160.0°), respectively. Additionally, it is worth noting that water plays an essential role in mediating the interaction between EPK and the β5 subunit. The interaction between the ketone oxygen with the side chain of Ser189 and the carboxyl group of Tyr228 is bridged by a structural water molecule (W3046), and the interaction between the oxygen at the C8-position of the main chain and the nitrogen of the isoxazole ring with Ala108 and Ala109 is bridged by another structural water molecule (W3027).

Interestingly, the docking result is consistent with the CoMFA contour map analysis, which further validates the 3D-QSAR model overall. No amino acid residues appeared upon the area of the benzene ring, indicating that bulky substituents in this position are favored for inhibitor activity. While the area above the isoxazole ring is occupied by residues of Ala109, Asp110, Cys111 and Gly107, suggesting that bulky substituents in this position will conflict with these residues and decrease the inhibitor activity. Similarly, the area beside the epoxyketone is occupied by residues Thr60, Arg78 and Ser189, where large substituents are also not favored. The electrostatic contour map can also be validated by the docking study. Due to the H-bond formed by the hydrogen at the N7-position of the main chain and backbone carbonyl group of Thr80, where the hydrogen is a hydrogen donor and Thr80 is a hydrogen receptor, negative charged substituents in this area is disfavored for inhibitor activity, while in the interaction bridged by W3027, where the oxygen at the C8-position of the main chain and the nitrogen of the isoxazole ring act as H-bond receptor, the backbone NH of Ala108 and Ala109 act as hydrogen donor, hence, positive charged substituents around the isoxazole are favored for inhibitor activity.

#### 3.4.2. TBA

**Figure 9.**
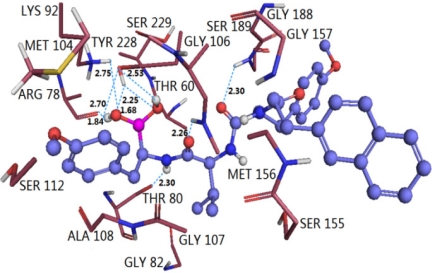
Docked conformations derived for molecule 2 of TBA (shown in ball and stick model) with the β5 subunit of human proteasome. H-bonds formed between residues and molecule are shown as dotted lines with blue color. The nonpolar hydrogen atoms are removed for clarity.

The binding mode of compound 2 shown in Figure 9 is taken as a docking model for analysis. The ligand core is anchored in the binding site by nine H-bonds contacting the protein. The two oxygen atoms at the C7- and C4-positions of the main chain form two H-bonds with the backbone NH of Ser189 (−O···HN, 2.30 Å, 134.3°) and backbone NH of Gly106 (−O···HN, 2.26 Å, 137.2°), respectively. While the two hydroxyl oxygen atoms at B1-position of the main chain, acting as hydrogen acceptors, form two H-bonds with the same hydroxyl hydrogen of Thr60 (–O···HO, 1.68 Å, 115.4°) and (–O···HO, 2.25 Å, 116.2°), respectively. The hydroxyl oxygen of Thr60 forms two H-bonds with the two hydroxyl hydrogen atoms at the B1-position of the main chain (−O···HO, 2.70 Å, 53.4°) and (−O···HO, 2.53 Å, 95.2°), respectively. Another hydroxyl oxygen of Thr80 forms a H-bond with hydrogen at the N3- position of the main chain (-O···HN, 2.30 Å, 154.8°), the carbonyl oxygen of Arg78 forms a H-bond with one hydroxyl hydrogen at the B1-position (-O···HO, 1.84 Å, 163.9°), meanwhile, the oxygen of this hydroxyl forms another H-bond with the backbone NH of Lys92 (-O···HN, 2.75 Å, 95.6°).

Similarly, we found that docking result is in agreement with CoMSIA maps overall. Residues of Ser189, Gly188 and Gly157 occupied the left area of the anisole group of the main chain, thus large substituents in this area conflict with these residues and are not favored for molecular activity. In contrary, no residues exist upon this anisole group, indicating that bulk substituents are favored in this region. These findings and the steric contour map validate each other well. For the electrostatic countour map, one big blue polyhedron beside the anisole group at the C2-position and two small ones adjacent to the methyl group of the anisole group at the C8- position and around the ethyl group at the C11-position of the main chain can be explained by the facts that two hydroxyl oxygen atoms at the B1-position of the main chain are H-bond acceptors in this area, where electropositive substituents are good to increase the activity of molecules. Similarly, a red polyhedron behind the B1-position of the main chain can also be interpreted by the two hydrogen atoms at the B1-position of the main chain as hydrogen bond acceptors, where electropositive substituents are favored. A series of hydrophobic residues Tyr288, Ala105, Ser229, Gly188 and Gly157 in the upper regions of the middle part of the molecule may interact with this part through hydrophobic interactions, suggesting that adding hydrophobic substituents in this region may increase the inhibitor activity. However, Thr80 and Gly106 residues interact with the ligand through H-bonds interaction; thus, more hydrophobic residues surrounding this area will decrease inhibitor activity. To the H-bond acceptor counter map, two hydroxyl oxygen atoms at the B1-position, acting as hydrogen acceptors, are involved in hydrogen bonding interactions with the backbone of Lys93 and side chain of Thr60. Thus, both the red and magenta polyhedrons are observed nearby the hydroxyl groups at the B1-position of the main chain, suggesting that a negatively charged substituent with hydrogen bond accepting capacity added to this position would engage in interactions with the donor and enhance the inhibitory activity.

### 3.5. Comparison of Binding Modes of Each Class

**Figure 10.**
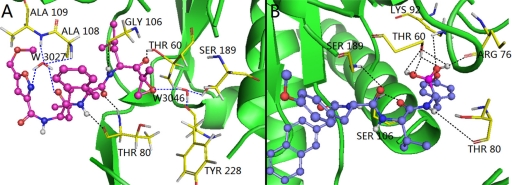
Stereo view of the docked conformations with compounds 11 and 2 in the active site of the β5 subunit. The H-bonds formed between residues and molecule directly and mediated by water indirectly are shown as dotted lines with black and blue color, respectively. Compounds 11 and 2, colored with magenta and blue, are presented in (**A**) and (**B**). The important amino acid residues, Thr60, Arg78, Thr80, Lys92, Ser106, Ala108, Ala109, Ser189 and Tyr228 and water molecules (stick rendering) are colored by atom type (C, yellow; N, blue; H, white; O, red). The nonpolar hydrogen atoms are removed for clarity.

The binding modes of these two types of PIs were compared on purpose to explore their similarities and differences and to get a better understanding of the variations in their biological activities. Based on the docking study, we found that H-bond and water-mediated interactions are both important between the EPK inhibitors and the target receptor. For EPK (shown in Figure 8), four H-bonds are formed between compound 11 and residues Thr60 (-O···HN, 1.78 Å, 131.9°), Thr80 (2.01 Å, 152.4°; 1.85 Å, 160.0°) and Gly106 (1.69 Å, 146.5°). Two water molecules (W3027 and W3046) mediated interactions are formed between compound 11 and residues Ser189, Tyr288, Ala108 and Ala 109. As regards TBA (shown in Figure 9), H-bond is vital to interactions between TBA inhibitors and the target receptor. Nine H-bonds are formed between compound 2 and residues Thr60 (2.53 Å, 95.2°; 2.25 Å, 116.2°; 2.70 Å, 53.4°; 1.68 Å, 115.4°), Arg78 (1.84 Å, 163.9°), Thr80 (2.30 Å, 154.8°), Lys92 (2.75 Å, 95.6°), Gly106 (2.26 Å, 137.2°) and Ser189 (2.30 Å, 134.3°). Among these, four H-bonds are concerned with the hydroxyl groups at the B1-position, which further confirmed that this structure is crucial to the peptide boronates PIs inhibitory activities. By comparison (shown in Figure 10), we obtained the following conclusions: (1) Thr60 (N-terminal Threonine) is important for these two series of PIs, which is in fully consistent with the literatures reported; (2) Common residues Thr80 and Gly106 are both involved in the binding modes. Therefore, these two residues are very important for the interaction between EPK/TBA and the β5 subunit; (3) Both EPK and TBA inhibitors form more than four H-bonds with the β5 subunit, indicating that they exhibit potent inhibitory activity; (4) Except the H-bond, the interaction mediated by water is also vital for EPK.

### 3.6. MD Simulations Analysis

When predicting the posing of a ligand into a protein, docking can provide a good starting point for further calculations with the aim of evaluating the stability of the predicted interactions involved in binding [66]. In order to investigate the positional and conformational changes of inhibitors relative to the active pocket and reveal the binding stability, two 5 ns MD simulations were further performed based on the aforementioned docking complex structures considering the effects of the receptor flexibility and the explicit water solvation.

#### 3.6.1. EPK

**Figure 11.**
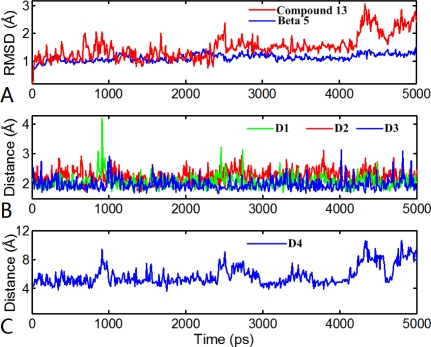
The results of molecular dynamics (MD) simulations. (**A**) The MD simulation time *versus* root mean-square deviation (RMSD, in Å) of the backbone atoms (C, N, and Cα) for the β5 subunit (blue) and compound 11 (red). (**B**) H-bond distance during MD simulations (D1:ligand@N7-H···NH of Thr80; D2: ligand@C5-O···HN of Thr80; D3: ligand@N4-H···O of Gly106). (**C**) Distance between the N-cap and the N atom of Ala109 during MD simulations (D4).

The results from the MD study are showed in Figure 11A. As we see, the overall average structure of the β5 subunit is conserved in time during simulations, which is reflected by the RMSD value (ranging from 0.7 to 1.3 Å), indicating the acceptability of this model. Interestingly, we found that the compound 11 (ligand) undergoes a conformational change during the MD simulations reflected by its RMSD value, which slowly jumped from 1.2 to 1.5 Å at 2.4 ns, and then jumped from 1.5 to 2.3 Å at 3.8 ns. To further investigate the differences between these conformations, three representative conformations of compound 11 are chosen from the corresponding phases (in 1450 ps, 3490 ps and 4840 ps, respectively) for further analyses.

**Figure 12.**
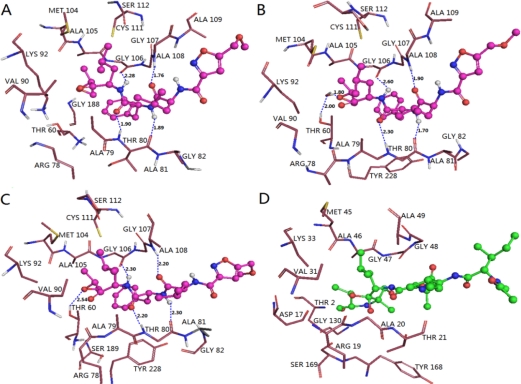
Different conformations derived for molecule 11 (shown in ball and stick with magenta color) with the β5 subunit of human proteasome in MD simulations: (**A**) 1450 ps; (**B**) 3490 ps; and (**C**) 4840 ps. (**D**) The conformation derived for EPX (shown as ball and sticks with green color) with template protein. H-bonds are shown as dotted lines with blue color. The nonpolar hydrogen atoms are removed for clarity.

Figure 12A depicts the early phase conformation of compound 11. During this period, three H-bonds are reserved from the docking results: the oxygen at the C5-position of the main chain formed a H-bond with the backbone NH of Thr80 (–O···HN, 1.90 Å, 143.7°); the two hydrogen atoms at N4- and N7-position of the main chain formed two H-bonds with the backbone carboxyl groups of Gly106 (-NH···O, 2.28 Å, 121.3°) and Thr80 (-NH···O, 1.89 Å, 150.3°), respectively. In addition, a new and stable H-bond is formed between the oxygen at the C8-position of the main chain and the backbone NH of Ala108 (–O···HN, 1.76Å, 166.2°). Additionally, the two original structural water molecules presenting in the docking structure moved away during simulations and the interactions mediated by these two waters also vanished. So, without the interactions, the N-cap ((5-MeOCH_2_)-3-isoxazole) of compound 11 rotated nearly 60° compared with the starting docking structure. Figure 12B depicts the transition state conformation of compound 11. In this complex structure, the above mentioned four H-bonds still exist, but the H-bond distances and angles are changed. Furthermore, another new two H-bonds are formed between the hydrogen at N7-position of the main chain and the ketone oxygen mediated by a water molecule. This conformation is similar to the former conformation. Figure 12C depicts the final conformation of compound 11. In this stage, five H-bonds are formed in the binding site. In addition to the aforementioned four H-bonds, a new H-bond is formed between the ketone oxygen and the side chain of Thr60 (–O···HO, 2.54 Å, 127.9°), which is different from the docking results. Since the three H-bonds appear in both docking and A, B, C conformations, the variation of these H-bonds distances are detected (shown in Figure 11B) during MD simulations. We found that the three H-bonds exist in nearly all simulation process with small distances (<3 Å), indicating that these H-bonds are important to stabilize the interactions between compound 11 and the β5 subunit. VMD software displayed that the N-cap of compound 11 moves severely. So, in order to track this change, the distance between the C* ( 
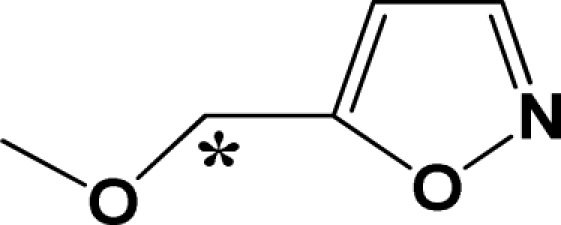
) atom of the N-cap and the N atom of Ala109 main chain is measured (shown in Figure 11C). By comparison with Figure 11A (red curve), we found that the variation trend of N-cap is similar to that of compound 11. From this, we can come to the conclusion that the conformational change of compound 11 mainly results from the movement of the N-cap, which could be explained by the binding mode that the N-cap extending to the outer free space, but other parts of this ligand are kept tightly in the inner through H-bond interactions.

Interestingly, we found that the final conformation of compound 11 has identical molecular orientation with the conformation of EPX extracted from the template 1G65_K (shown in Figure 12D). Therefore, the obtained results of MD simulations of the protein-ligand system suggest that this class of PIs adopts the same conformation to interact with the β5 subunit. Although the ligand undergoes several movements to yield different conformations (from conformation A to C), the binding site is always same during the whole dynamic process.

#### 3.6.2. TBA

**Figure 13.**
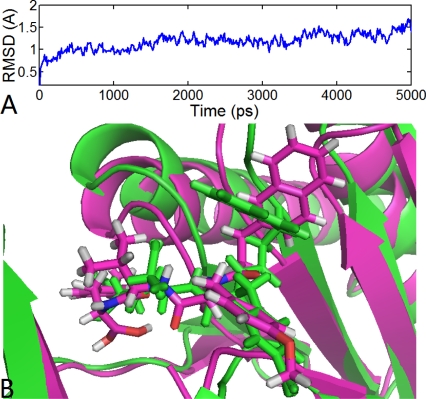
(**A**) RMSD plot of docked complex *versus* the MD simulation time in the MD-simulated structures. (**B**) View of superimposed backbone atoms of the average structure of the MD simulation (green) and the initial structure (magenta) for compound 2. Compound 2 is shown as sticks in green for the average structure and in magenta for initial complex.

**Figure 14.**
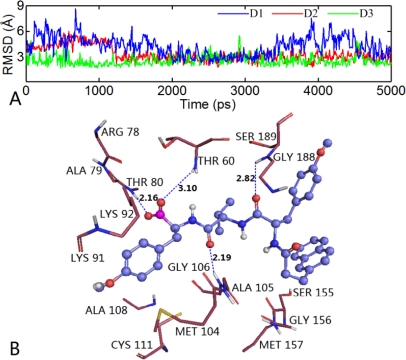
(**A**) H-bond distance during MD simulations (D1:ligand@B-OH···NH of Thr60; D2: ligand@N7-O···HN of Ser189; D3: ligand@C4-O···O of Gly106); (**B**) The conformation derived for compound 2 (shown in ball and stick with blue color) with the β5 subunit of human proteasome in MD simulations. H-bonds formed by residues and molecule are shown as dotted lines with blue color. The nonpolar hydrogen atoms are removed for clarity.

By VMD software, we find that the conformation of compound 2 changed slightly overall during the dynamic process. Thus, the RMSD values of the compound 2-β5 subunit system as a whole with respect to the original structure of the docking complex are analyzed (shown in Figure 13A). We can see that the RMSD values of this system range from 0.6 to 1.7 Å, and are relatively stable after 1500 ps with the RMSD of about 1.2 Å, suggesting that the molecular systems behaved well thereafter. The low RMSD fluctuations confirm the feasibility of the binding poses predicted by Surflex dock. A superposition of the average structure of the ensemble for the last 100 ps and the docked structure is shown in Figure 13B. It is noted that there is no significant difference between the average structure extracted from MD simulations and the docked model of the complex, except the anisole group rotated 64°, showing the rationality and stability of the docking model. In order to explore the similarities and differences between the results of docking and MD simulation, the interactions between compound 2 and the β5 subunit was analyzed. From Figure 14B, we can see that four H-bonds formed during MD simulations. Among these four H-bonds, three formed at the same sites as those in docking mode with different distances and angles. The two oxygen atoms at the C7- and C4-positions of the main chain formed two stable H-bonds with the backbone NH of Ser189 (-O···HN, 2.82 Å, 121.1°) and backbone NH of Gly106 (-O···HN, 2.19 Å, 158.2°), respectively; the hydroxyl oxygen at the B1-position of the main chain formed a weak H-bond with the back bone NH of Thr60 (-O···HN, 3.10 Å, 144.6°). A new H-bond is formed between the other hydroxyl oxygen at the B1-position of the main chain and Thr80 (-O···HN, 2.16 Å, 166.7°). Similarly, we also detected variations of the three H-bond distances during the simulation process (shown in Figure 14A).

Generally speaking, the conformations obtained after simulations are more stable and credible than the docked conformations. This can be elucidated by the fact that the docking method has some intrinsic drawbacks when considering that the effect of solvating water molecules is not explicitly treated. Nevertheless, MD simulation is carried out closer to the physiological environment conditions. Thus, compared with the docking analysis, the corresponding binding modes from MD simulations have a better correlation with the 3D-QSAR analysis.

## 4. Conclusion

In this study, the ligand-based and receptor-based 3D-QSAR studies using CoMFA and CoMSIA methods have been performed on two kinds of PIs. The 3D-QSAR studies involving Dragon-descriptors yielded stable and statistically significant predictive models with relatively high Q^2^, small SEE and high R_pre_^2^ values. Overall, for EPK, the study of its optimal model reveals that both steric and electrostatic fields and molecular descriptors EEig04r, Mor24e are critical to its inhibitory activities, and TBA optimal model implies that the electrostatic, hydrophobic fields and Dragon-descriptors, RDF050M and AlogP2 are more important to its inhibitory activities. Satisfyingly, the interactions identified from the 3D contour maps are in good correlation with the specific interactions between the inhibitors and the amino acid residues identified in the docked binding structures. Residues Thr60, Thr80, Gly106 and Ser189 are demonstrated to be vital in facilitating β5 subunit recognition of its inhibitors. MD simulation results of compound 11-β5 subunit complex revealed that EPK inhibitors undergo a conformational change to interact with the receptor, which is consistent with the crystal structure (PDB code: 1G65_K). In general, the good consistency between the 3D-QSAR, docking and MD results further implies the robustness of the established 3D-QSAR models. Therefore, the obtained models can be used in guiding future structural modifications and synthesizing novel potent inhibitors against the β5 subunit of human proteasome.

## Figures and Tables

**Table 1.** Summary of the CoMFA and CoMSIA results.

	**EPK**				**TBA**			
	
**parameters**	**Ligand-based**		**Receptor-based**		**Ligand-based**		**Receptor-based**	
	
	CoMFA	CoMSIA	CoMFA	CoMSIA	CoMFA	CoMSIA	CoMFA	CoMSIA
N	6	3	4	3	3	3	2	2
Q^2^	0.462	0.409	0.503	0.467	0.601	0.622	0.580	0.560
SEP	0.478	0.477	0.445	0.453	0.282	0.275	0.284	0.291
SEE	0.202	0.339	0.129	0.188	0.218	0.208	0.246	0.232
F	45.479	25.021	176.631	105.373	30.974	34.777	32.774	38.905
R^2^_pred_	0.820	0.479	0.628	0.648	0.773	0.821	0.804	0.766
Field contributions %								
S	0.418	0.046	0.278	0.117	0.240	0.035	0.102	0.066
E	0.429	0.176	0.526	0.355	0.068	0.117	0.059	0.123
D		0.157		0.252				
H		0.143				0.122	0.327	
A		0.107				0.078	0.512	0.109
EEig04r	0.100	0.264	0.133	0.210				
Mor24e	0.053	0.107	0.063	0.066				
RDF050M					0.239	0.213	0.080	0.291
AlogP2					0.453	0.435	0.181	0.412

N is the optimal number of components; Q^2^ is the leave-one-out (LOO) cross-validation coefficient;

SEP is standard error of prediction; SEE is the standard error of estimation; F is the F-test value;

R^2^_pred_ is the predictive correlation coefficient; S, E, H, D, A is the steric, electrostatic, hydrophobic, as well as hydrogen-bond donor and acceptor fields, respectively.

**Table 2.** Symbols of the descriptors used in the models and their definitions.

**Symbols**	**Descriptor family**	**Definition**
EEig04r	Topological	Eigenvalue 04 from edge adj. matrix weighted by resonance integrals
Mor24e	3D-MoRSE	3D-MoRSE-signal 24/weighted by atomic Sanderson electronegativities
RDF050M	RDF	Radial Distribution Function-5.0/weighted by atomic masses
AlogP2	Molecular properties	Squared Ghose-Crippen octanol-water partition coeff. (logP^2)

## Appendix

### Appendix 1. EPK structures and their chymotrypsin-like (CT-L) inhibitory activity.

**Table:**

**Compd.**	**structure**	**IC_50_(nM)**	**pIC_50_**
1		5.7	8.2441
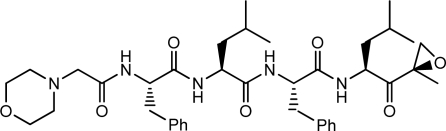
2		1000	6.000
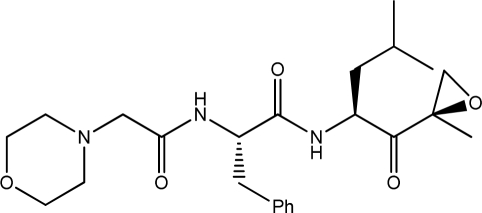

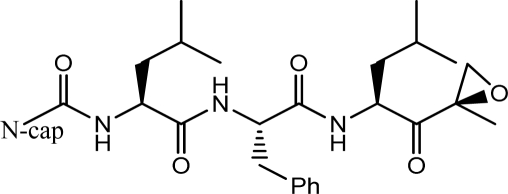

**Compd.**	**N-cap**	**IC_50_(nM)**	**pIC_50_**
3	Morpholine-CH_2_	57	7.2441
4	3-furan	34	7.4685
5^b^	2-thiophene	10	8.0000
6	5-oxazole	27	7.5686
7	5-isoxazole	5.8	8.2366
8	3-isoxazole	7.4	8.1308
9	(5-Me)-3-isoxazole	1.8	8.7447
10	(5-iPr)-3-isoxazole	6.3	8.2007
11	(5-MeOCH_2_)-3-isoxazole	1.2	8.9208
12	3-pyrazole	36	7.4437
13^b^	2-imidazole	107	6.9706
14	(N-Me)-3-pyrazole	76	7.1192
15^b^	(N-Me)-2-imidazole	122	6.9136
16	(5-Me)-3-pyrazole	38	7.4202
17	4-pyridine	20	7.6990
18	4-pyridazine	12	7.9208
19	2-pyrazine	752	6.1238
20	2-(R)-tetrahydrofuran	34	7.4685
21	2-(S)-tetrahydrofuran	42	7.3768
22	(5-Me)-3- isoxazole-NH	13	7.8861

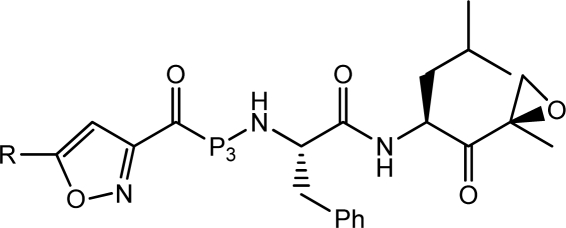

**Compd.**	**R**	**P_3_**	**IC_50_(nM)**	**pIC_50_**
23	1-(1,2,4)-triazole-CH_2_	Leu	13	7.8861
24^b^	1-imidazole-CH_2_	Leu	15	7.8239
25^b^	N-Me-piperazine-CH_2_	Leu	94	7.0269
26	Me	Leu	4.1	8.3872
27	Morpholine-CH_2_	Ser(OMe)	9.3	8.0315
28	Me	Ser(OMe)	5.7	8.2441
29	Me	(4-Thiazolyl)-ala	6.7	8.1739
30	Morpholine-CH_2_	(4-Thiazolyl)-ala	6.9	8.1611
31	Me	(2-Py)-ala	13	7.8861
32	Morpholine-CH_2_	(2-Py)-ala	5.2	8.284
33^b^	Me	(3-Py)-ala	1.6	8.7959
34^b^	Morpholine-CH_2_	(3-Py)-ala	6.4	8.1938
35^b^	Me	(4-Py)-ala	2.8	8.5528
36	Morpholine-CH_2_	(4-Py)-ala	5.1	8.2924

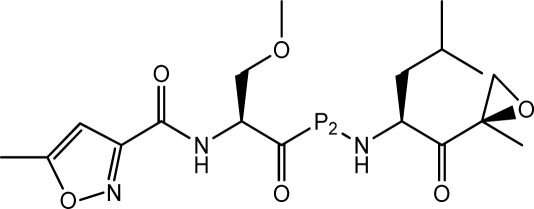

**Compd.**	**P_2_**	**IC_50_(nM)**	**pIC_50_**
37	cyhxy-ala	13	7.8861
38	3-thienyl-ala	9.2	8.0362
39	4-thiazolyl-ala	16	7.7959
40	Val	44	7.3565
41	Leu	20	7.6990
42	Ala	19	7.7212
43	Abu	54	7.2676
44^b^	CN-Ala	40	7.3979
45	Ser(OMe)	11	7.9586

Note:

^b^ compounds used in the test set.

### Appendix 2. TBA structures and their chymotrypsin-like (CT-L) inhibitory activity.

**Table:**

**Compd.**	**R**	**IC_50_(μM)**	**pIC_50_**
1^b^		0.019	7.7212
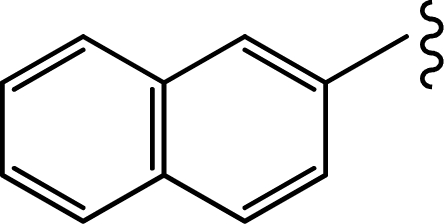
2		0.022	7.6576
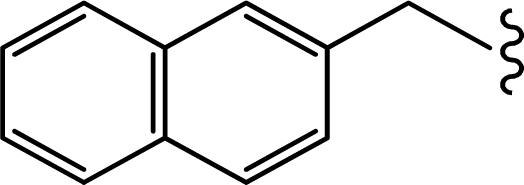
3		0.0041	8.3872
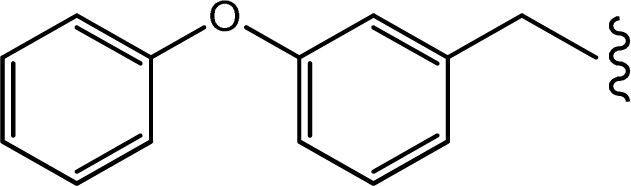
4	1-isoquinolyl	0.041	7.3872
5	3-isoquinolyl	0.059	7.2291
6	6-isoquinolyl	0.38	6.4202
7	2-quinolyl	0.10	7.0000
8^b^	3-quinolyl	0.056	7.2518
9^b^	4-quinolyl	0.049	7.3098
10	8-quinolyl	0.093	7.0315
11	pyrazyl	0.24	6.6198
12	2-pyridyl	0.23	6.6383
13^b^	3-pyridyl	0.50	6.3010
14	4-pyridyl	2.3	5.6383

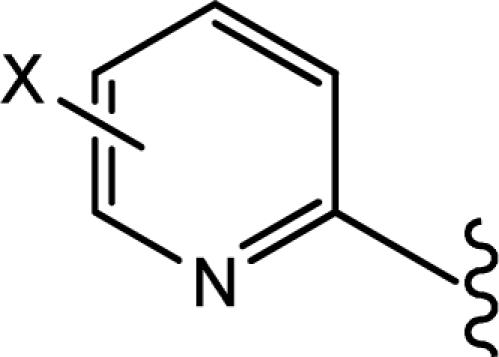

**Compd.**	**X**	**IC_50_(μM)**	**pIC_50_**
15	3-Me	0.085	7.0706
16	4-Me	0.14	6.8539
17	5-Me	0.12	6.9208
18	6-Me	0.088	7.0555
19	3-F	0.14	6.8539
20^b^	5-F	0.081	7.0915
21	6-F	0.11	6.9586
22	3-Cl	0.083	7.0809
23	4-Cl	0.088	7.0555
24	5-Cl	0.083	7.0809
25	6-Cl	0.10	7.0000
26	3,6-Cl_2_	0.053	7.2757
27	3-Br	0.061	7.2147
28	4-Br	0.095	7.0223
29	5-Br	0.093	7.0315
30	6-Br	0.092	7.0362
31^b^	4-CF_3_	0.15	6.8239
32	5-CF_3_	0.11	6.9586
33	3-HO	0.39	6.4089
34	6-HO	0.24	6.6198
35^b^	3-MeO	0.13	6.8861
36	4-MeO	0.087	7.0605
37	5-MeO	0.094	7.0269
38	6-MeO	0.059	7.2291
39	4-Me_2_N	0.11	6.9586

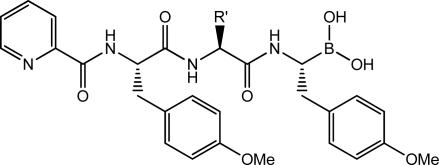

**Compd.**	**R**′	**IC_50_(μM)**	**pIC_50_**
40^b^	H	0.019	6.5850
41	Me	0.039	6.9586

Note:

^b^ compounds used in the test set.

## References

[1] De Bettignies G, Coux O (2010). Proteasome inhibitors: Dozens of molecules and still counting. Biochimie.

[2] Wehenkel M, Ho YK, Kim K-B (2009). Proteasome inhibitors: Recent progress and future directions. Modul. Protein Stab. Cancer Ther.

[3] Golab J, Bauer TM, Daniel V, Naujokat C (2004). Role of the ubiquitin-proteasome pathway in the diagnosis of human diseases. Clin. Chim. Acta.

[4] Griffin TA, Nandi D, Cruz M, Fehling HJ, Van Kaer L, Monaco JJ, Colbert RA (1998). Immunoproteasome assembly: Cooperative incorporation of interferon γ (IFN-γ)-inducible subunits. J. Exp. Med.

[5] Kisselev AF, Goldberg AL (2001). Proteasome inhibitors: From research tools to drug candidates. Chem. Biol.

[6] Groll M, Ditzel L, Löwe J, Stock D, Bochtler M, Bartunik HD, Huber R (1997). Structure of 20S proteasome from yeast at 2.4 Å resolution. Nature.

[7] Löwe J, Stock D, Jap B, Zwickl P, Baumeister W, Huber R (1995). Crystal structure of the 20S proteasome from the Archaeon T. acidophilum at 3.4 Å resolution. Science.

[8] Groll M, Berkers CR, Ploegh HL, Ovaa H (2005). Crystal structure of the boronic acid-based proteasome inhibitor bortezomib in complex with the Yeast 20S proteasome. Structure.

[9] Unno M, Mizushima T, Morimoto Y, Tomisugi Y, Tanaka K, Yasuko N, Tsukihara T (2002). Structure determination of the constitutive 20S Proteasome from bovine liver at 2.75 Å resolution. J. Biochem.

[10] Jung T, Catalgol B, Grune T (2009). The proteasomal system. Mol. Aspect. Med.

[11] Rivett AJ (1989). The multicatalytic proteinase: Multiple proteolytic activities. J. Biol. Chem.

[12] Chen P, Hochstrasser M (1996). Autocatalytic subunit processing couples active site formation in the 20S proteasome to completion of assembly. Cell.

[13] Kisselev AF, Akopian TN, Castillo V, Goldberg AL (1999). Proteasome active sites allosterically regulate each other, suggesting a cyclical bite-chew mechanism for protein breakdown. Mol. Cell.

[14] Baumeister W, Walz J, Zuhl F, Seemuller E (1998). The proteasome: Paradigm of self-compartmentalizing protease. Cell.

[15] Groll M, Bajorek M, Kohler A, Moroder L, Rubin DM, Huber R, Glickman MH, Finley D (2000). A gated channel into the proteasome core particle. Nat. Struct. Biol.

[16] Jäger S, Groll M, Huber R, Wolf DH, Heinemeyer W (1999). Proteasome β-type Subunits: Unequal roles of propeptides in core particle maturation and a hierarchy of active site function. J. Mol. Biol.

[17] Lam YA, Pockart CM, Alban A, Landon M, Jamieson C, Ramage R, Mayer RJ, Layfield R (2000). Inhibition of the ubiquitin-proteasome system in Alzheimer’s disease. PANS.

[18] Ding Q, Keller JN (2001). Proteasome and proteasome inhibiton in the central nervous system. Biol. Med.

[19] Marfella R, D’Amico M, Esposito K, Baldi A, Di Filippo C, Siniscalchi M, Sasso FC, Portoghese M, Cirillo F, Cacciapuoti F (2006). The ubiquitin-proteasome system and inflammatory activity in diabetic atherosclerotic plaques. Diabetes.

[20] Xu J, Wu Y, Zhang M, Wang S, Zou MH (2007). Proteasome-dependent degradation of guanosine 5′-triphosphate cyclohydrolase I causes tetrahydrobiopterin deficiency in diabetes mellitus. Circulation.

[21] Wojcik C, Di Napoli M (2004). Ubiquitin-proteasome system and proteasome inhibition: New strategies in sroke therapy. Stroke.

[22] Wu WKK, Cho CH, Lee CW, Wu K, Fan D, Yu J, Sung JJ (2010). Proteasome inhibition: A new therapeutic strategy to cancer treatment. Cancer Lett.

[23] Hideshima T, Chauhan D, Richardson P, Mitsiades C, Mitsiades N, Hayashi T, Munshi N, Dang L, Castro A, Palombella, Adams J, Anderson KC (2002). NF-kappa B as a therapeutic target in multiple myeloma. J. Biol. Chem.

[24] Lopes UG, Erhardt P, Yao R, Cooper GM (1997). p53-Dependent induction of apoptosis by proteasome inhibitors. J. Biol. Chem.

[25] Pleban E, Bury M, M1ynarczuk I, Wójcik C (2001). Effects of proteasome inhibitor PSI on neoplastic and non-transformed cell lines. Folia Histochem. Cytobiol.

[26] Adams J, Kauffman M (2004). Development of the proteasome inhibitor VelcadeTM (Bortezomib). Cancer Investig.

[27] Adams J (2002). Proteasome inhibition: A novel approach to cancer therapy. Trends Mol. Med.

[28] Richardson PG, Briemberg H, Jagannath S, Wen PK, Barlogie B, Berenson J, Singhal S, Siegel DS, Irwin D, Schuster M (2006). Frequency, characteristics, and reversibility of peripheral neuropathy during treatment of advanced multiple myelomawith bortezomib. J. Clin. Oncol.

[29] Sugawara K, Hatori M, Nishiyama Y, Tomita K, Kamei H, Konishi M, Oki T (1990). Eponemycin, a new antibiotic active against B16 melanoma. I. Production, isolation, structure and biological activity. J. Antibiot.

[30] Hanada M, Sugawara K, Kaneta K, Toda S, Nishiyama Y, Tomita K, Yamamoto H, Konishi M, Oki T (1992). Epoxomicin, a new antitumor agent of microbial origin. J. Antibiot.

[31] Meng L, Mohan R, Kwok BHB, Elofsson M, Crews CM (1999). Epoxomicin, a potent and selective proteasome inhibitor, exhibits *in vivo* antiinflammatory activity. Med. Sci.

[32] Groll M, Kim KB, Kairies N, Huber R, Crews CM (2000). Crystal structure of epoxomicin: 20S proteasome reveals a molecular basis for selectivity of α’, β’-epoxyketone proteasome inhibitors. J. Am. Chem. Soc.

[33] Elofsson M, Splittgerber U, Myung J, Mohan R, Crews CM (1999). Towards subunit-specific proteasome inhibitors: Synthesis and evaluation of peptide alpha’, beta’-epoxyketones. Chem. Biol.

[34] Momose I, Sekizawa R, Hashizume H, Kinoshita N, Homma Y, Hamada M, Iinuma H, Takeuchi T (2001). Tyropeptins A and B, new proteasome inhibitors produced by Kitasatospora sp. MK993-dF2. J. Antibiot.

[35] Watanabe T, Abe H, Momose I, Takahashi Y, Ikeda D, Akamatsu Y (2010). Structure-activity relationship of boronic acid derivatives of tyropeptin: Proteasome inhibitors. Bioorg. Med. Chem. Lett.

[36] Liao SY, Chen JC, Qian L, Shen Y, Zheng KC (2008). QSAR studies and molecular design of phenanthrene-based tylophorine derivatives with anticancer activity. QSAR Comb. Sci.

[37] Wei SP, Ji ZQ, Zhang HX, Zhang JW, Wang YH, Wu WJ (2010). Isolation, biological evaluation and 3D-QSAR studies of insecticidal/narcotic sesquiterpene polyol esters. J. Mol. Model.

[38] Wang X, Yang W, Xu X, Zhang H, Wang Y (2010). Studies of benzothiadiazine derivatives as Hepatitis C Virus NS5B polymerase inhibitors using 3D-QSAR, molecular docking and molecular dynamics. Curr. Med. Chem.

[39] Zhu Y-Q, Pei J-F, Liu Z-M, Lai L-H (2006). 3D-QSAR studies on tripeptide aldehyde inhibitors of proteasome using CoMFA and CoMSIA methods. Bioorg. Med. Chem.

[40] Zhu Y-Q, Lei M, Lu A-J, Zhao X, Yin X-J, Gao Q-Z (2009). 3D-QSAR studies of boron-containing dipeptides as proteasome inhibitors with CoMFA and CoMSIA methods. Europ. J. Med. Chem.

[41] Zhou H-J, Aujay MA, Bennett MK, Dajee M, Demo SD, Fang Y, Ho MN, Jiang J, Kirk CJ, Laidig GJ, Lewis ER, Lu Y, Muchamuel T, Parlati F, Ring E, Shenk KD, Shields J, Shwonek PJ, Stanton T, Sun CM, Sylvain C, Woo TM, Yang J (2009). Design and synthesis of an orally bioavaliable and selective peptide epoxyketone proteasome inhibitor (PR-047). J. Med. Chem.

[42] Johnsamuel J, Byun Y, Jones TP, Endo Y, Tjarks W (2003). A new strategy for molecular modeling and receptor-based design of carborane containing compounds. J. Organometal. Chem.

[43] Li Y, Wang Y-H, Ding J, Wang Y, Chang Y-Q, Zhang S-W (2009). In silico prediction of androgenic and nonandrogenic compounds using random forest. QSAR Comb. Sci.

[44] Wang Y-H, Li Y, Yang S-L, Yang L (2005). An in silico approach for screening flavonoids as P-glycoprotein inhibitors based on a Bayesian-regularized neural network. J. Comput. Aid Mol. Design.

[45] Wang Y-H, Li Y, Yang S-L, Yang L (2005). Classification of substrates and inhibitors of p-glycoprotein using unsupervised machine learning approach. J. Chem. Inf. Model.

[46] Wang Y-H, Li Y, Li Y-H, Yang S-L, Yang L (2005). Modeling Km values using electrotopological state: Substrates for cytochrome P450 3A4-mediated metabolism. Bioorg. Med. Chem. Lett.

[47] Todeschini R, Consonni V (2000). Handbook of Molecular Descriptors.

[48] Cramer RD, Patterson DE, Bunce JD (1988). Comparative molecular field analysis (CoMFA). 1. Effect of shape on binding of steroids to carrier proteins. J. Am. Chem. Soc.

[49] Tetko IV, Tanchuk VY, Villa AE (2001). Prediction of n-octanol/water partition coefficients from PHYSPROP database using artificial neural networks and E-state indices. J. Chem. Inf. Comput. Sci.

[50] Schafferhans A, Klebe G (2001). Docking ligands onto binding site representations derived from proteins built by homology modelling. J. Mol. Biol.

[51] Thompson JD, Higgins DG, Gibson TJ (1994). CLUSTAL W: Improving the sensitivity of progressive multiple sequence alignment through sequence weighting, position-specific gap penalties and weight matrix choice. Nucleic Acids Res.

[52] Arnold K, Bordoli L, Kopp J, Schwede T (2006). The SWISS-MODEL workspace: A web-based environment for protein structure homology modeling. Bioinformatics.

[53] Schwede T, Kopp J, Guex N, Peitsch MC (2003). SWISS-MODEL: An automated protein homology-modeling server. Nucleic Acids Res.

[54] Guex N, Peitsch MC (1997). SWISS-MODEL and the Swiss-PdbViewer: An environment for comparative protein modeling. Electrophoresis.

[55] Jain AN (2003). Surflex: Fully automatic flexible molecular docking using a molecular similarity-based search engine. J. Med. Chem.

[56] Case DA, Darden TA, Cheatham I, Simmerling CL, Wang J, Duke RE, Luo R, Crowley M, Walker RC, Zhang W (2008). AMBER 10.

[57] Wang J, Wolf RM, Caldwell JW, Kollman PA, Case DA (2004). Development and testing of a general amber force field. J. Comput. Chem.

[58] Jakalian A, Jack DB, Bayly CI (2002). Fast, efficient generation of high-quality atomic charges. AM1-BCC model: II. Parameterization and validation. J. Comput. Chem.

[59] Hummer G, Rasaiah JC, Noworyta JP (2001). Water conduction through the hydrophobic channel of a carbon nanotube. Nature.

[60] Jorgensen WL, Chandrasekhar J, Madura JD, Klein ML (1983). Comparison of simple potential functions for simulating liquid water. J. Chem. Phys.

[61] Essmann U, Perera L, Berkowitz ML, Darden T (1995). A smooth particle mesh Ewald method. J. Chem. Phys.

[62] Ryckaert JP, Ciccotti G, Berendsen HJC (1977). Numerical integration of the cartesian equations of motion of a system with constraints: Molecular dynamics of n-alkanes. J. Comput. Phys.

[63] Hammer MC, Steinhauer V, Gasteiger J (1999). Deriving the 3D structure of organic molecules from their infrared spectra. Vib. Spectrosc.

[64] Cavasotto CN, Phatak SS (2009). Homology modeling in drug discovery: Current trends and applications. Drug Discov. Today.

[65] Zhang B, Li Y, Zhang H, Ai C (2010). 3D-QSAR and molecular docking studies on derivatives of MK-0457, GSK1070916 and SNS-314 as inhibitors against Aurora B Kinase. Int. J. Mol. Sci.

[66] Lavecchia A, Cosconati S, Novellino E, Calleri E, Temporini C, Massolini G, Carbonara G, Fracchiolla G, Loiodice F (2007). Exploring the molecular basis of the enantioselective binding of penicillin G acylase towards a series of 2-aryloxyalkanoic acids: A docking and molecular dynamics study. J. Mol. Graph. Model.

